# Effect of Biomolecular Conformation on Docking Simulation: A Case Study on a Potent HIV-1 Protease Inhibitor

**Published:** 2015

**Authors:** Nima Razzaghi-Asl, Saghi Sepehri, Ahmad Ebadi, Ramin Miri, Sara Shahabipour

**Affiliations:** a *Department of Medicinal Chemistry, School of Pharmacy, Ardabil University of Medical Sciences, Ardabil, Iran.*; b *Drug and Advanced Sciences Research Center, School of Pharmacy, Ardabil University of Medical Sciences, Ardabil, Iran.*; c *Department of Medicinal Chemistry, Faculty of Pharmacy, Isfahan University of Medical Sciences.*; d *Department of Medicinal Chemistry, School of Pharmacy, Hamadan University of Medical Sciences, Hamadan, Iran.*; e *Medicinal and Natural Products Chemistry Research Center, Shiraz University of Medical Sciences, Shiraz, Iran.*; f *Department of Medicinal Chemistry, School of Pharmacy, Shiraz University of Medical Sciences, Shiraz, Iran.*

**Keywords:** AIDS, HIV-1 PR, Conformational variation, Amprenavir, Docking

## Abstract

Human immunodeficiency virus infection/acquired immunodeficiency syndrome (HIV/AIDS) is a disease pertained to the human immune system. Given its crucial role in viral replication, HIV-1 protease (HIV-1 PR) is a prime therapeutic target in AIDS therapy. In this regard, the dynamic aspects of ligand-enzyme interactions may indicate an important role of conformational variability in HIV-1 PR inhibitor/drug design. In the present contribution, the effect of HIV-1 PR flexibility (within multiple crystallographic structures of HIV-1 PR) on binding to the Amprenavir was elucidated via an ensemble docking approach. Molecular docking studies were performed via advanced AutoDock4.2 software. Ensemble docking of Amprenavir into the active site of various conformations of HIV-1 PR predicted different interaction modes/energies. Analysis of binding factors in terms of docking false negatives/positives revealed a determinant role of enzyme conformational variation in prediction of optimum induced fit (PDB ID: 1HPV). The outcomes of this study demonstrated that conformation of receptor may significantly affect the accuracy of docking/binding results in structure-based rational design of anti HIV-1 PR agents. Furthermore; some strategies to re-score the docking results in HIV-1 PR targeted docking studies were proposed.

## Introduction

Acquired immunodeficiency syndrome (AIDS) is a disease related to the human immune system (1). Human immunodeficiency virus (HIV) has been identified as the etiological agent of AIDS (2). Cells of the immune system, called T-cells or CD4 cells that are responsible for fighting against infections and other physiological disturbances are attacked and destroyed by HIV. One of the essential HIV enzymes, whose activity is necessary for viral replication, is HIV-1 protease (HIV-1 PR) (3). In fact, production of mature and infectious viral particles is depended on the proteolytic activity of the HIV-1 PR and for this reason; this enzyme was recognized as a major therapeutic target in AIDS therapy and has been the subject of numerous drug design studies (4, 5). 

HIV-1 PR inhibitors are believed to inactivate the HIV-1 protease leading to the immature, non-infectious viral particles (6). Most of the developed HIV-1 protease inhibitors are peptidomimetic molecules (7). The main drawback of peptidomimetic compounds is their low oral bioavailability arising from high molecular weight and poor solubility (8). Due to this limitation, many researchers have focused on nonpeptidic HIV-1 PR inhibitors (3, 9, 10).

Amprenavir, Atazanavir, Darunavir, Indinavir, Fosamprenavir, Lopinavir, Nelfinavir, Ritonavir, Saquinavir and Tipranavir are typical anti-AIDS drugs that have been approved by the United States Food and Drug Administration (US FDA) as HIV-1 PR inhibitors. These drugs are currently used in combination therapy with reverse transcriptase inhibitors (11, 12). Although several successful drugs have been developed against AIDS, current status shows a rapid emergence of drug resistance to most of the HIV-1 PR inhibitors (11). In this regard, recent research aimed at proposing new anti-protease agents with minimum side effects and being able to delay the appearance of resistance (13, 14).

**Figure 1:**
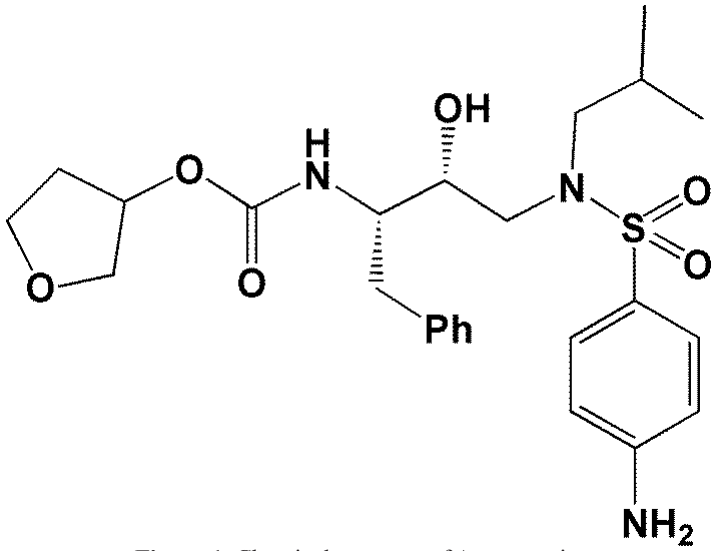
Chemical structure of Amprenavir

In continuation to our interest in structure based modeling of bioactive molecules (15, 16) and to further elucidate the important role of target conformation in molecular docking results, we decided to explore the significance of HIV-1 PR flexibility through ensemble docking of Amprenavir into the multiple crystallographic structures of HIV-1 proteases (17, 18). Amprenavir (Figure 1) is a potent and selective HIV-1 PR inhibitor with sub-nanomolar HIV-1 PR inhibition activity (k_i_=0.6 nM) (19, 20) and hence was selected as a model in our studies.

## Experimental

*Materials and methods*

All the required holo/apo PDB structures were retrieved from the Brookhaven protein databank (http://www.rcsb.org). Flexible-ligand docking studies were performed using AutoDock4.2 program (21). All 3D structures of ligands were prepared using CORINA server (http://www.molecular-networks.com/). The pre-processing steps for receptor crystallographic files (PDB codes: 2PQZ, 2Q5K, 3EKV, 3MXD, 3O9F, 3O9I, 3SA5, 3SA8, 3SAB, 4DJP, 4DQB, 2PSU, 2Q54, 3EKX, 3MXE, 3O9G, 3SA3, 3SA6, 3SA9, 3SAC, 4DJQ, 2PSV, 2Q55, 3EM3, 3NLS, 3O9H, 3SA4, 3SA7, 3SAA, 4DJO, 4DJR, 1AJV, 1BWA, 1CPI, 1HPV, 1T3R, 1XL5, 1A8G, 1A9M, 1BWB, 1AJX, 1BV9, 1DIF, 1GNO, 1IDB, 1MUI, 1T7J, 1XL2, 2I0A, 2I0D and 3IXO) were performed within AutoDock Tools 1.5.4 program (ADT) and WHAT IF server (http://swift.cmbi.ru.nl/servers/html/) (21, 22). The 3D structure of HIV-1 PR enzyme with the code 1HPV (including Amprenavir ligand) was used as a reference point in our docking studies. All hydrogens were properly added to the receptor PDB files using WHAT IF server. ADT program was used to merge non-polar hydrogens into related carbon atoms of the receptor and Kollman charges were also assigned. For docked ligands, non-polar hydrogens were merged; Gasteiger charges assigned and torsions degrees of freedom were also allocated by ADT program. 100 independent genetic algorithm (GA) runs were considered. 2.5×10^7 ^maximum number of evaluations was used for Lamarckian GA method. All other docking parameters were set at their default values. A grid of 60×60×60 points in x, y, and z direction was built centered on the center of mass of the catalytic site of HIV-1 PR crystallographic structures. Cluster analysis was performed on the docked results using a root mean square (RMS) tolerance of 2 A˚. 

Schematic 2D representations of the ligand-receptor interactions were all generated using LIGPLOT (23).

## Results

*Docking validation*

A performance of a docking simulation method was checked via its ability in reproducing a binding mode for a co-crystallographic (cognate) ligand (24). For this purpose, the structure of a cognate ligand (Amprenavir) was retrieved and re-docked into the active site of HIV-1 PR structures. Root mean square deviations (RMSD) of the Cartesian coordinates of the re-docked ligand atoms proved the validation of docking method for further modeling studies (Table 1) (25). As it is obvious from the summarized data, all the crystallographic files under study represented adaptable predictability level (26) within 100 independent genetic algorithm (GA) runs and 2.5×10^7 ^maximum number of evaluations for Lamarckian GA method. It should be noted that those structures exhibiting RMSD values over 3 may also pass the filter when considering their number of active torsions (27). 

**Table 1:** Docking validation results for different *holo* PDB structures of MAO-B using AutoDock4.2

**PDB code**	**GA runs**	**Maximum No. of energy evaluations**	**RMSD from reference structure (Å)**
4DJO	100	2.5×10^7^	3.53
4DJP	100	2.5×10^7^	3.17
4DJQ	100	2.5×10^7^	3.49
4DJR	100	2.5×10^7^	2.20
4DQB	100	2.5×10^7^	2.73
3SA7	100	2.5×10^7^	3.17
3SA8	100	2.5×10^7^	2.61
3SA9	100	2.5×10^7^	3.37
3NLS	100	2.5×10^7^	2.36
3O9G	100	2.5×10^7^	2.63
3O9F	100	2.5×10^7^	3.76
3O9H	100	2.5×10^7^	2.99
3O9I	100	2.5×10^7^	3.05
3SA3	100	2.5×10^7^	3.03
3SA4	100	2.5×10^7^	2.26
3SA5	100	2.5×10^7^	1.60
3SA6	100	2.5×10^7^	2.34
3SAC	100	2.5×10^7^	1.14
2Q5K	100	2.5×10^7^	3.13
2Q54	100	2.5×10^7^	4.16
2Q55	100	2.5×10^7^	3.33
3EM3	100	2.5×10^7^	2.39
3EKV	100	2.5×10^7^	1.53
3EKX	100	2.5×10^7^	2.30
3MXD	100	2.5×10^7^	3.00
3MXE	100	2.5×10^7^	2.89
2PSV	100	2.5×10^7^	2.49
2PQZ	100	2.5×10^7^	0.34
2PSU	100	2.5×10^7^	2.94
1T3R	100	2.5×10^7^	2.56
1MUI	100	2.5×10^7^	2.97
2I0A	100	2.5×10^7^	2.85
1IDB	100	2.5×10^7^	2.02
1T7J	100	2.5×10^7^	0.47
1XL2	100	2.5×10^7^	2.91
1XL5	100	2.5×10^7^	2.28
1GNO	100	2.5×10^7^	3.85
1A9M	100	2.5×10^7^	3.45
1DIF	100	2.5×10^7^	4.45
1AJV	100	2.5×10^7^	1.18
1AJX	100	2.5×10^7^	0.64
1CPI	100	2.5×10^7^	1.72
1BWA	100	2.5×10^7^	2.06
1BWB	100	2.5×10^7^	1.56
1BV9	100	2.5×10^7^	2.13
3SAB	100	2.5×10^7^	2.28
3SAA	100	2.5×10^7^	1.00
2I0D	100	2.5×10^7^	2.15
1HPV	100	2.5×10^7^	1.80
3IXO a	-	-	-

a 3IXO is an apo file.

*Ensemble docking of Amprenavir*

We aimed to evaluate the Amprenavir / HIV-1 PR interaction considering ligand induced enzyme conformation. Our dataset included one apo and fifty holo HIV-1 PR structures. These structures were subjected to ensemble docking procedure. Crystallographic structure of the Amprenavir/HIV-1 PR complex was deposited in the PDB website (1HPV) (28) and as mentioned before, this crystallographic structure was considered as the reference point in our docking simulations. 

**Figure 2:**
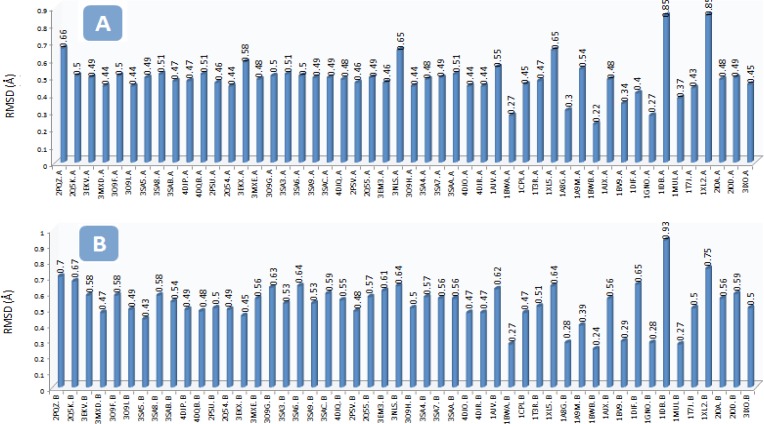
The RMSD (Ǻ) of the backbone carbon atoms (C*α*) in the A) chain A of HIV-1 PR and B) chain B of HIV-1 PR structures with regard to the Amprenavir/HIV PR complex (PDB code: IHPV) To run the project, Amprenavir was docked into the active site of multiple HIV-1 PR conformations. AutoDock binding affinities and Amprenavir binding conformations (ligand binding ensembles) are represented in Table 2 and Figure 3, respectively.

The RMSD of the backbone carbon atoms (C*α*) in the selected PDB structures ranged 0.22–0.85 and 0.24-0.93 Å in chains A and B of HIV-1 PR, respectively (with regard to the PDB code: IHPV; Figure 2). Different RMSD values indicated the conformational changes of HIV-1 PR upon binding to the various inhibitors.

**Table 2:** AutoDock binding affinities of Amprenavir/HIV-1 PR complexes

**PDB code of the receptor**	**AutoDock binding energy (kcal/mol)**	**PDB code of the receptor**	**AutoDock binding energy (kcal/mol)**
1A8G	-7.28	3EKX	-7.95
1A9M	-8.30	3EM3	-8.90
1AJV	-7.77	3MXD	-7.86
1AJX	-7.24	3MXE	-7.66
1BV9	-7.38	3NLS	-8.24
1BWA	-9.25	3O9F	-8.42
1BWB	-8.67	3O9H	-7.81
1CPI	-7.41	3O9I	-7.59
1DIF	-6.64	3O9G	-7.37
1GNO	-8.60	3SA3	-7.90
1HPV	-7.83	3SA4	-7.92
1IDB	-6.90	3SA5	-7.97
1MUI	-7.09	3SA6	-7.72
1T3R	-8.11	3SA7	-7.51
1T7J	-7.45	3SA8	-8.11
1XL2	-6.73	3SA9	-7.87
1XL5	-7.60	3SAA	-9.15
2I0A	-7.88	3SAB	-8.16
2I0D	-7.37	3SAC	-8.06
2PQZ	-7.32	4DJO	-7.85
2PSU	-7.66	4DJP	-7.46
2PSV	-8.12	4DJQ	-7.79
2Q5K	-8.00	4DJR	-7.67
2Q54	-8.19	4DQB	-7.85
2Q55	-7.33	3IXO	-4.94
3EKV	-8.10		

**Figure 3:**
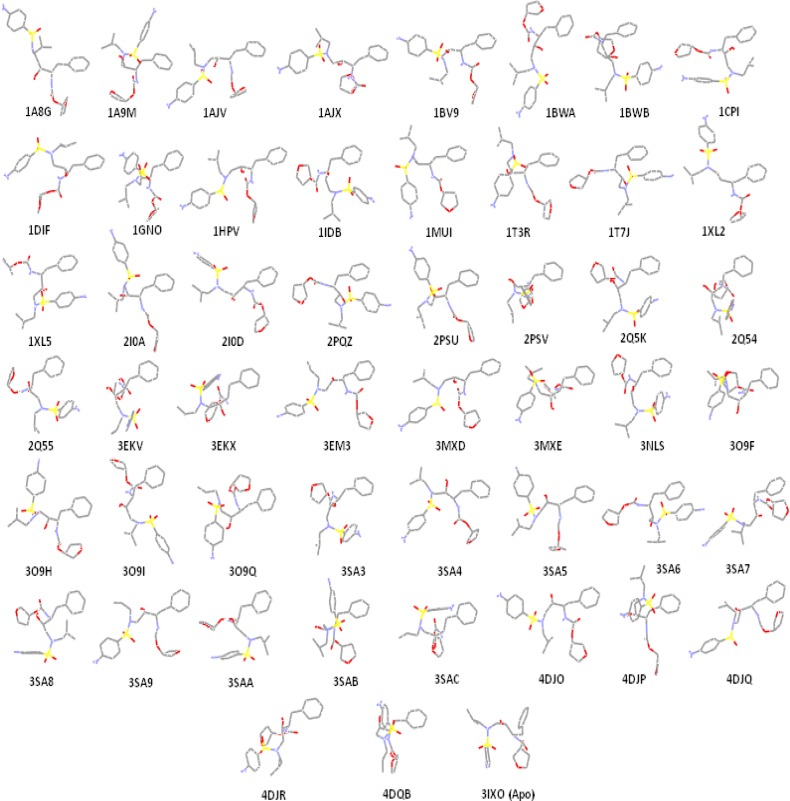
Amprenavir binding ensembles as the result of docking into the different conformations of the HIV-1 PR; each conformation of the target is designated by its relevant PDB code

*Amprenavir/HIV-1 PR interactions*

**Figure 4:**
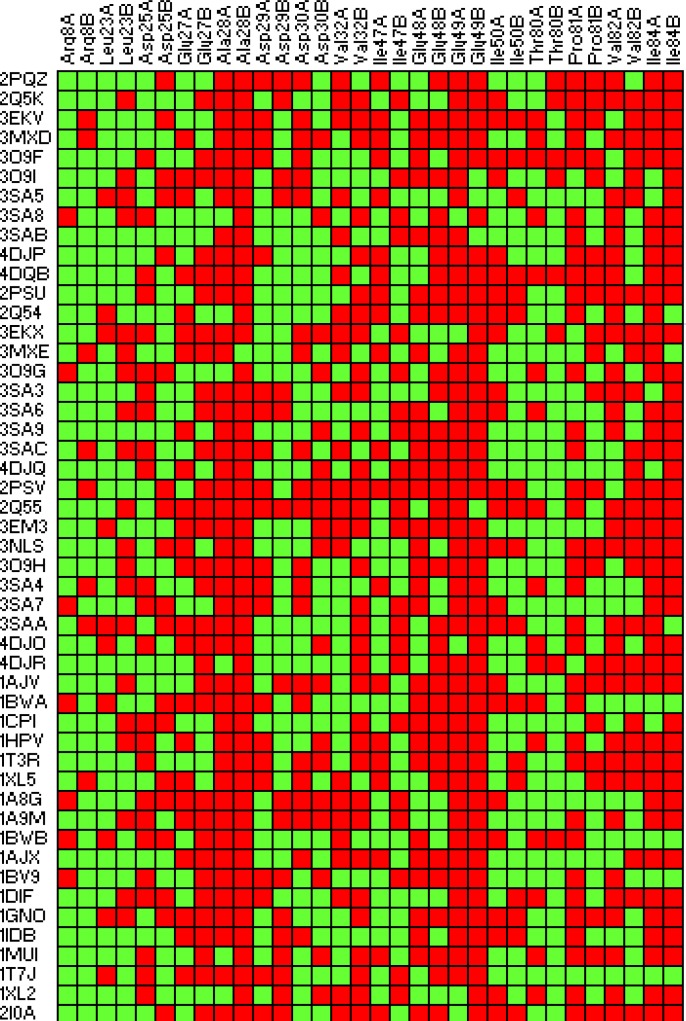
2D fingerprint representation of lipophilic contacts in binding of Amprenavir to the HIV-1 PR ensembles. Red: interacted; Green: Non-interacted

Lipophilic contacts (Figure 4) and H-bond interactions (Table 3) in docked Amprenavir-protease complexes were monitored. According to the 2D Ligplot diagrams, thirty-two residues of the HIV-1 PR were found to make lipophilic contacts with Amprenavir within fifty-one enzyme conformational structures. In the case of hydrogen bond interactions, a total of nineteen amino acids interacted Amprenavir within 51 conformations of the enzyme. Data are summarized in Table 3 while numbers refer to the H-bond distances. 

**Table 3:** Possible H-bond interactions of Amprenavir with different conformations of the HIV-1 PR

	**Ile50(B)**	**Gly48(B)**	**His48(B)**	**Asp29(B)**	**Asp30(B)**	**Asp25(B)**	**Asp25(A)**	**Il50(A)**	**Asp29(A)**	**Gly27(A)**	**Gly27(B)**	**Asp30(A)**	**Thr80(A)**	**Arg8(A)**	**Leu50(B)**	**Leu50(A)**	**Val82(A)**	**Arg8(B)**	**Gly48(A)**
2PQZ	2.12						2.14	1.91											
2Q5K	2.07	2.48			2.21	1.69						2.37							
3EKV					1.83		1.81			2.08									
3MXD	2.4					2.24		2.36											
3O9F				1.98						1.87							3.12		
3O9I					2.24		1.9		1.99										
3SA5							2.67	2.05	3.01		2.19								
3SA8	1.96			1.85				2.01		1.88	2.18								
3SAB	2.34			2.04		1.74		2.08			2.04	2.28							
4DJP	2.26						1.78			1.98		2.12							
4DQB					2.01	2.26			2.42			2.06							
2PSU					2.31	1.81				1.99		2.37							
2Q54				2.4	1.9	2.61	1.86				2.12	2.13							
3EKX	2.28			2.34		2.39, 2.4													
3MXE	2.19						2.25	1.93	1.98										
3O9G	1.96							1.88		2.02	2.20								
3SA3					2.05	1.84				2.09		4.9							
3SA6	2.35				1.99	2.04				2.32									
3SA9	2.22					1.86		2.16			2.36	2.41		2.04					
3SAC	2.42					2.81		2.12											
4DJQ	2.31			2.04		1.86		1.86			2.18								
2PSV							2.34			2.13									
2Q55							2.46												
3EM3				1.94		1.75						2.33	2.15		2.05	2.06			
3NLS				1.9			1.99				2.32	2.02							
3O9H	2.43					1.71		2.09					2.71				2.77	2.43	
3SA4	1.94					2.4		2.24		2.03							2.12		
3SA7	2.49	1.91		2.67	2.14						2.18								
3SAA							2.33												1.94
4DJO	2.15			1.96		2.21		2.35						2.58					
4DJR	1.92			1.89		2.14		1.75											
1AJV	2.32					1.94													
1BWA				2.79			2.7												
1CPI	1.84							2.17		1.85	1.81								
1HPV	2.11					2.34		2.31				2.28							
1T3R						1.83		2.27		2.38			2.23						
1XL5							1.97	2.29, 2.21			2.08								
1A8G	2.19	2.10, 1.91																	
1A9M	2.32		2.22	2.22	1.89	2.07		2.41											
1BWB	2.11																		
1AJX	2.26				2.32		2.2	2.2	2.13										
1BV9	1.98					2.39		2.11		2.07									
1DIF								2.15				3.25							2.88
1GNO					1.81		1.96		1.94			1.99							
1IDB					2.04		1.78												
1MUI	2.04					1.77		2.38											
1T7J	1.82				2.12	1.92		2.17											
1XL2	2.34	1.95																	
2I0A					1.91		1.95			2.14		2.06							
2I0D	2.11	1.87		2.32	1.79			1.89											
Apo								2.53											

*Validation of virtual binding affinities*

**Figure 5:**
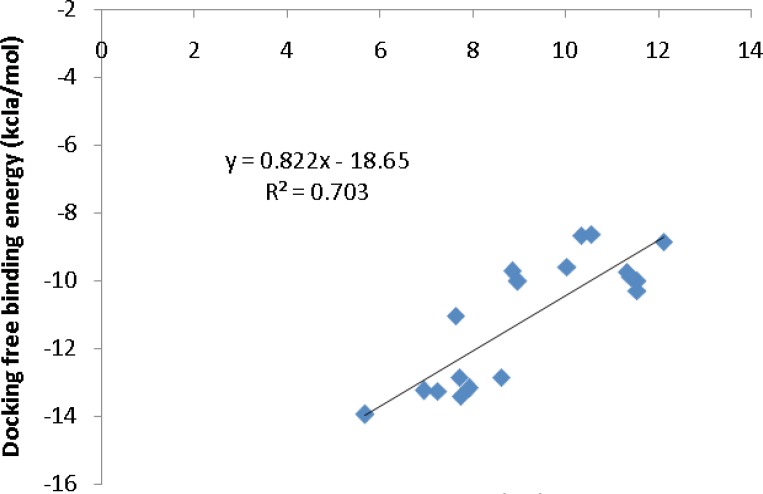
Correlation of AutoDock binding affinities and biological activities (PDB bind database) for a series of co-crystallographic HIV-1 PR inhibitors (PDB codes: 4DJO, 4DJP, 4DJQ, 4DJR, 2Q5K, 3MXD, 3MXE, 2PSV, 2PQZ, 2PSU, 2I0A, 1GNO, 1A9M, 1AJV, 1AJX, 1BWA, 1BV9, 2I0D

**Figure 6:**
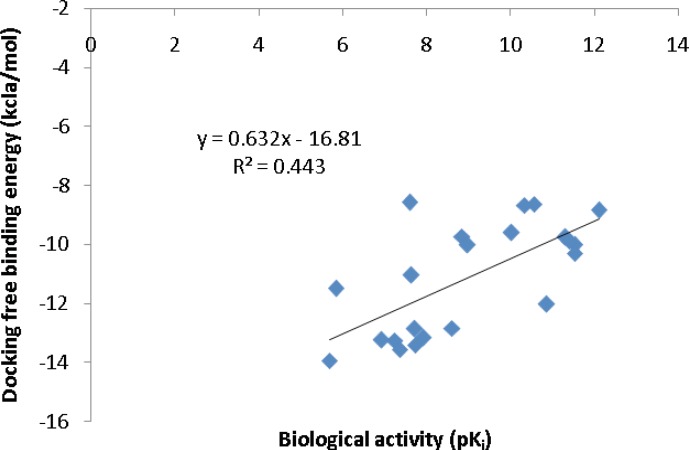
Correlation of AutoDock binding affinities and biological activities (Binding MOAD database) for a series of co-crystallographic HIV-1 PR inhibitors (PDB codes: 4DJO, 4DJP, 4DJQ, 4DJR, 2Q5K, 2Q5S, 3MXD, 3MXE, 2PSV, 2PQZ, 2PSU, 1T3R, 2I0A, 1XL2, 1XL5, 1GNO, 1A9M, 1AJV, 1AJX, 1BWA, 1BV9, 2I0D).

To further validate the AutoDock binding affinities, two co-crystallographic HIV-1 PR/inhibitor datasets with available biological activities at PDB bind (29) (Figure 5) or Binding MOAD (30) (Figure 6) databases were selected for a regression analysis. AutoDock binding affinities were all obtained from the self-docking step. 

*Effective factors in binding conformation*

**Figure 7:**
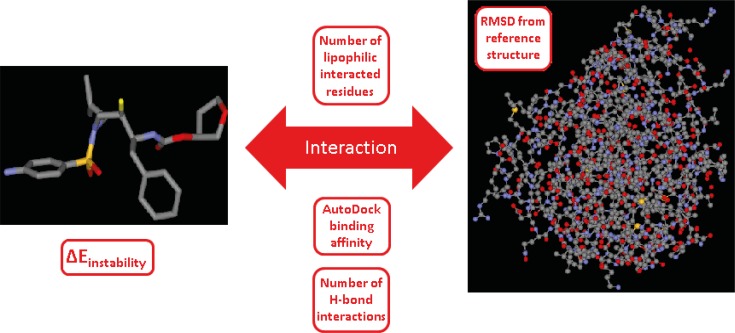
Schematic representation of the ligand-enzyme binding system and its related descriptors, system constituents in our study are ligand (Amprenavir), enzyme (HIV-1 PR) and their interaction. System descriptors are AutoDock score, protein deviation from apo form, number of lipophilic interacted residues, number of H-bond interactions and instability energy of ligand binding pose (∆E_instability_). ∆E_instability _indicates the instability gain of geometrically optimum conformation as the result of binding to the receptor (19).

We were interested in finding the factors that might be determinant in induced conformation of HIV-1 PR/Amprenavir complex (PDB ID: 1HPV). For these purpose; a binding system comprised of three major constituents (ligand, enzyme and their interaction) was taken into consideration. Such a system may be defined by several descriptors that are related to the system constituents (Figure 7).

To account for the conformational deviation of HIV-1 PR from its apo structure (native conformation), a pair wise structure alignment was done. RMSD (Ǻ) of the backbone carbon atoms (C*α*) in the chain A of individual HIV-1 PR structures with regard to the HIV PR apo structure (PDB ID: 3IXO) are reported in Table 4. 

**Table 4:** Estimated binding factors for various docked Amprenavir/HIV-1 PR systems

**Code of docked HIV-1 PR file**	**AutoDock binding affinity** **(kcal/mol)**	**Number of lipophilic interacted residues**	**Number of H-bond interactions**	**RMSD from reference structure ** b **(kcal/mol)**	**∆E**_instability _ c **of docked ligand conformation ** **(kcal/mol)**	
2PQZ	-7.32	17	3	0.70	74.63
2Q5K	-8.00	18	5	0.57	47.69
3EKV	-8.10	21	3	0.53	69.81
3MXD	-7.86	19	3	0.49	83.93
3O9F	-8.42	19	3	0.56	65.42
3O9I	-7.59	17	3	0.49	65.56
3SA5	-7.97	15	4	0.52	80.89
3SA8	-8.11	14	5	0.55	41.07
3SAB	-8.16	11	6	0.54	73.27
4DJP	-7.46	16	4	0.49	67.26
4DQB	-7.85	19	4	0.57	66.53
2PSU	-7.66	18	4	0.50	71.54
2Q54	-8.19	15	6	0.51	64.30
3EKX	-7.95	21	4	0.60	75.94
3MXE	-7.66	14	4	0.55	76.66
3O9G	-7.37	16	4	0.57	81.69
3SA3	-7.90	16	4	0.52	69.02
3SA6	-7.72	16	4	0.55	71.66
3SA9	-7.87	14	6	0.51	96.32
3SAC	-8.06	18	3	0.54	74.84
4DJQ	-7.79	13	5	0.52	89.37
2PSV	-8.12	23	2	0.48	75.17
2Q55	-7.33	23	1	0.54	86.89
3EM3	-8.90	17	6	0.52	76.43
3NLS	-8.24	18	4	0.69	84.52
3O9H	-7.81	17	6	0.48	75.92
3SA4	-7.92	16	5	0.54	68.09
3SA7	-7.51	15	3	0.53	72.84
3SAA	-9.15	19	2	0.56	71.18
4DJO	-7.85	16	5	0.48	70.58
4DJR	-7.67	14	4	0.47	99.29
1AJV	-7.77	20	2	0.59	80.68
1BWA	-9.25	17	2	0.43	82.76
1CPI	-7.41	14	4	0.49	93.63
1HPV ^a^	-7.83	19	4	0.38	88.07
1T3R	-8.11	19	4	0.49	68.50
1XL5	-7.60	18	4	0.69	81.04
1A8G	-7.28	18	3	0.40	97.52
1A9M	-8.30	18	6	0.65	72.82
1BWB	-8.67	15	1	0.40	75.10
1AJX	-7.24	15	5	0.56	89.08
1BV9	-7.38	15	4	0.46	66.60
1DIF	-6.64	18	3	0.47	85.69
1GNO	-8.60	20	4	0.41	109.50
1IDB	-6.90	14	2	0.80	82.72
1MUI	-7.09	17	3	0.47	65.69
1T7J	-7.45	13	4	0.52	21.84
1XL2	-6.73	14	2	0.87	75.61
2I0A	-7.88	20	4	0.53	63.65
2I0D	-7.37	16	5	0.55	67.98

a *Crystallographic system comprising Amprenavir and HIV-1 PR*

b *Reference structure is the apo form of HIV-1 PR (PDB code: ****3IXO****). *

c *∆E *_*instability *_*= E*_*top-ranked docked *_*– E*_*optimized conformation*_

*Analysis of binding results via docking false negative/positives*

Ensemble docking approach may be interpreted in terms of predicted false negative (FN)/false positive (FP) results. High rate of false negatives/positives is a common issue in docking procedure leading to low “hit rates”. Due to this rationale, we decided to evaluate the docking results (Table 4) via FP and FN results.

**Figure 8:**
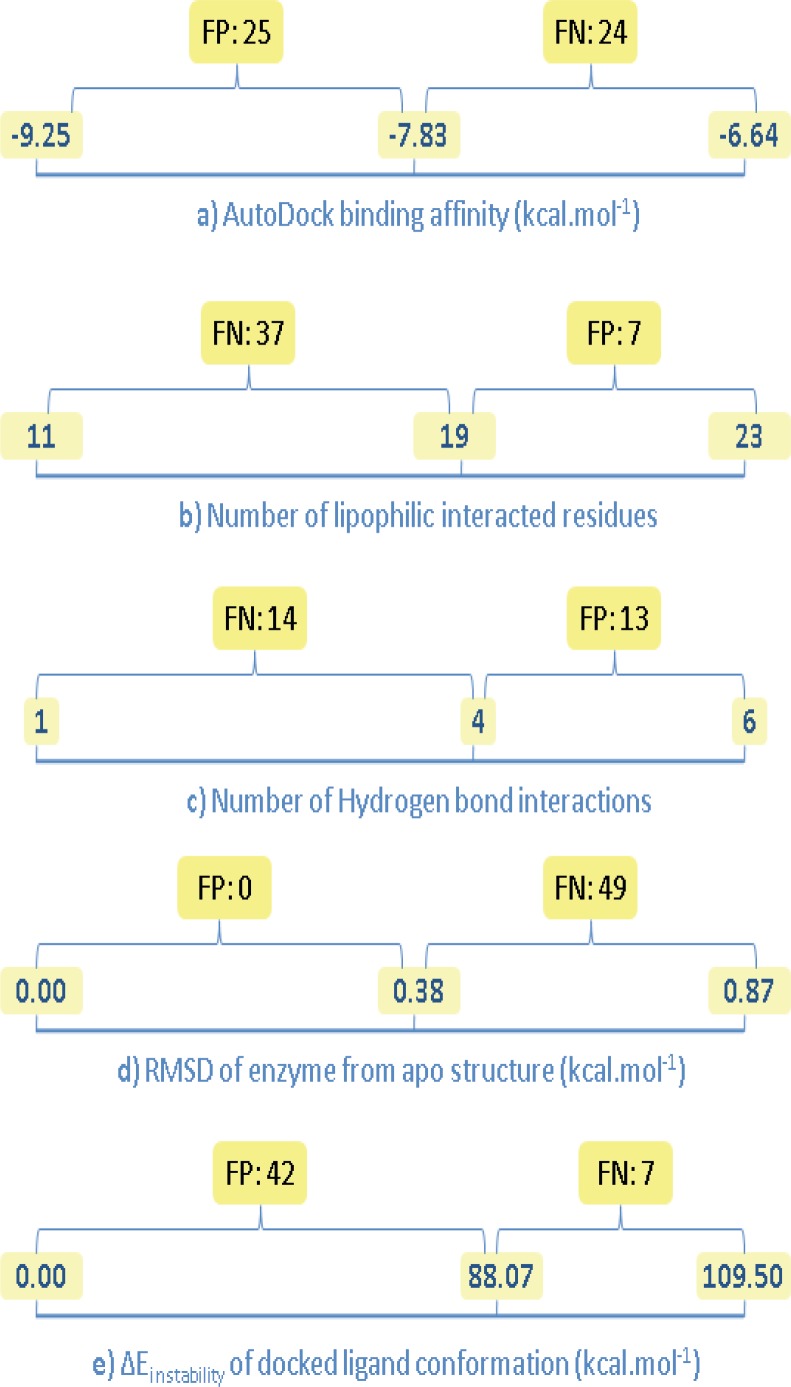
Number of false negative (FN) and false positive (FP) results for the estimated binding factors of docked Amprenavir/HIV-1 PR complexes; left side digits indicate optimum levels (except for b and c), center digits show the estimated value for Amprenavir/HIV-1 PR co-crystallographic complex, right side digits indicate non-optimum levels (except for a, d and e

For the sake of clarity, estimated descriptors (binding factors) for Amprenavir/HIV-1 PR co-crystallographic complex (IHPV) were considered as reference points in our analysis. In this manner, two distinct regions may be considered for each binding factor; a distance between reference level and optimum level including FPs and a distance between reference level and non-optimum level including FNs (Figure 8). 

## Discussion

*Ensemble docking approach*

Docking is a popular virtual structure-based method that is used in the design of biologically interesting molecules (31). It enables the prediction of stereoelectronic complementary fit of a potential bioactive ligand with its biomolecular target. In this regard; availability of crystallographic data on HIV-1 PR (Brookhaven protein databank website: http://www.rcsb.org) facilitated the performance of structure based drug discovery projects aiming at HIV-1 PR as a biomolecular target for AIDS disease. 

The HIV-1 PR consists of two identical 99 amino acid monomers representing a homodimer with C2 symmetry. Each subunit includes one of the two conserved triads (Asp-Thr-Gly) containing the catalytically active aspartate residues; Asp 25 and Asp 25′ (32). It has been well known that upon binding of different HIV-1 PR inhibitors, significant conformational changes might be expected for the enzyme (33, 34). Indeed, dynamic aspects of binding in the interaction of HIV-1 PR inhibitors with HIV-1 PR active site are crucially important for the design of novel enzyme inhibitors. Due to the computational cost in designating numerous degrees of freedom, incorporation of meaningful protein flexibility during a docking procedure is a difficult task although several efforts have been performed (35).

One of the alternative approaches for the flexible-receptor docking is the cross-docking of a typical ligand into the multiple crystallographic structures of the receptor (protein ensemble structures) (21). Holo crystallographic structures of targets provide appropriate models that represent real ligand induced conformations upon binding to the various chemical scaffolds (different inhibitors). In the case of biological targets lacking sufficient crystallographic holo structures, conformational ensemble may be generated virtually. However the advantage of the latter approach would be the possibility of generating more protein conformations but at the same time, a major drawback remains; the produced protein conformations may not be indicative of real structures. 

**Figure 9:**
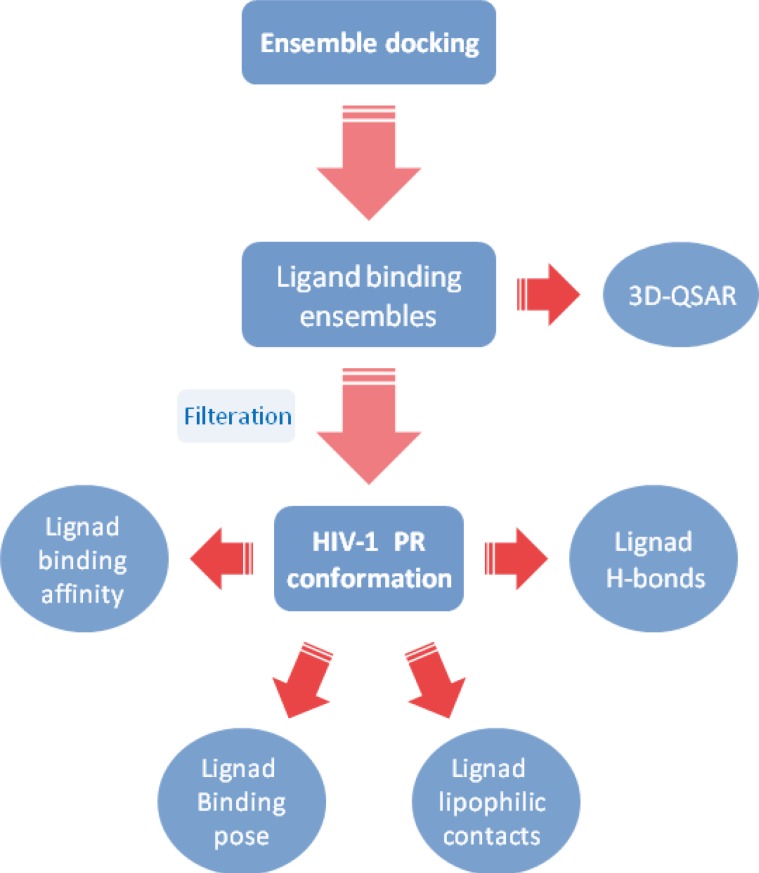
A typical flowchart representing an ensemble docking protocol for HIV-1 PR inhibitors

A simple flow chart representing the ensemble docking procedure might be depicted as below (Figure 9). It should be noted that ligand binding ensembles (resulted from ensemble docking) may be subsequently exploited as valuable input data for quantitative structure binding relationship studies.

Results of ensemble docking showed that Amprenavir interacted with HIV-1 PR active site via different binding modes. None of the docked ligands showed completely identical binding poses in the active site of the HIV-1 PR and the best scored conformation might not be supported with highest binding energy (refer to Table 2). 

*Amprenavir/HIV-1 PR Interactions*

**Figure 10:**
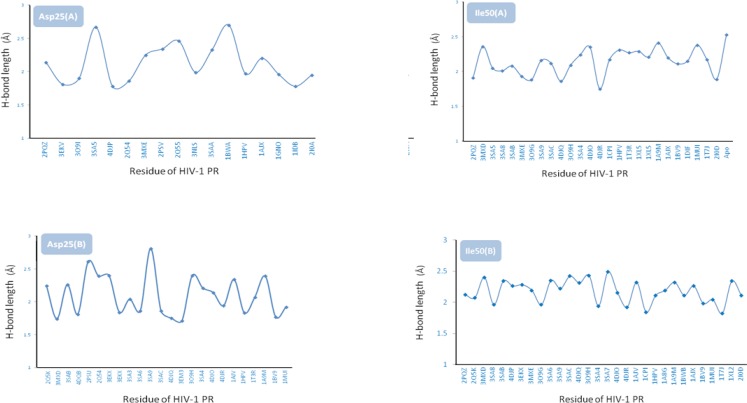
Fluctuation of H-bond lengths in binding of Amprenavir with Asp25(A), Ile50(A), Asp25(B) and Ile50(B) residues in HIV-1 PR crystallographic ensembles

The frequency of occurrence for a specific chemical interaction in multi-conformational ligand-enzyme assemblies may indicate the significance of such interaction in ligand-enzyme complex. Regarding the binding data, some principles might be driven:

- Docked Amprenavir showed different hydrophobic and H-bond binding patterns in multiple conformational ensembles of HIV-1 PR active site.
- Docking results demonstrated that Asp25(A), Ile50(A), Asp25(B) and Ile50(B) were determinant residues contributing to key H-bonds (with Amprenavir) within HIV-1 PR crystallographic ensembles. For more clarification, the fluctuation of H-bond lengths between these four residues and Amprenavir in the active site of HIV-1 PR ensembles is depicted in Figure 10. H-bond distance variations ranged 1.78-2.70, 1.75-2.53, 1.69-2.81 and 1.82-2.49 Å for Asp25(A), Ile50(A), Asp25(B) and Ile50(B) residues, respectively.

The frequency of occurrence for lipophilic interactions could be prioritized as Ala28(B) > Glu49(B) > Ala28(A)=Glu49(A) > Ile84(B) > Ile84(A) > Glu48(B) > Val32(B)=Pro81(A) > Glu27(B) > Val82(A) > Glu48(A) > Val82(B) > Asp25(A)=Glu27(A) > Ile50(A) > Asp30(A) > Val32(A) > Ile47(B) > Pro81(B) > Asp29(A) > Ile47(A) > Leu23(B) > Asp25(B) > Asp30(B) > Ile50(B) > Thr80(A) > Asp29(B) > Leu23(A) > Thr80(B) > Arg8(B) > Arg8(A). Capital letters in the parentheses indicate the chain of HIV-1 PR including the designated amino acid.

**Figure 11:**
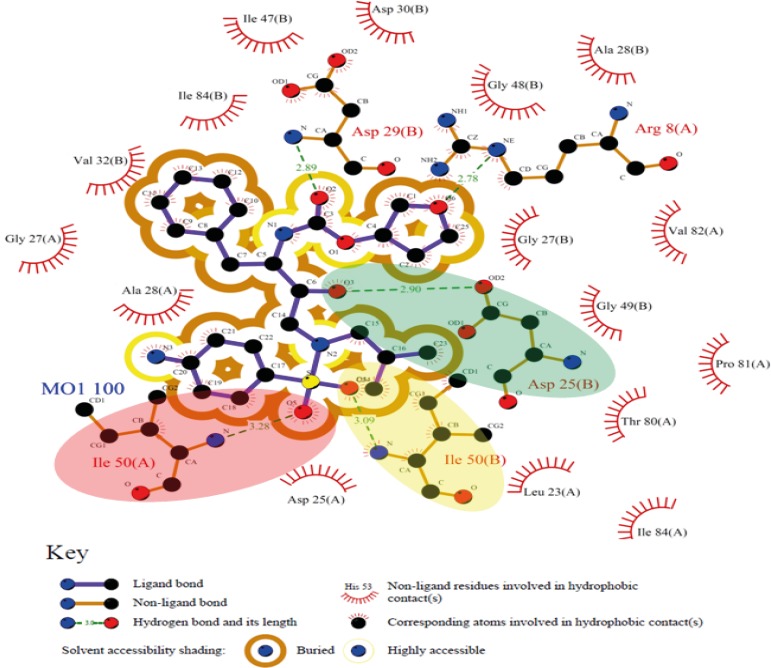
2D schematic representation of binding interactions between Amprenavir and HIV-1 PR active site (deposited PDB code: 4DJO), green, yellow and red ovals represent H-bonds between Amprenavir and Asp25(B), Ile50(B) and Ile50(A), respectively

- On the basis of binding results, Ala28(B) is the most important residue contributing to key electrostatic interactions in nearly all of the Amprenavir binding poses. Such priority orders emphasize the effect of target conformation on docking results.
- Comparison of different binding poses of Amprenavir revealed that the binding conformations represented by 2PSV and 2Q55 PDB codes were supported by the highest lipophilic contacts (Figure 4). For HIV-1 PR induced conformation designated by the PDB code 3SAB, minimum lipophilic contacts could be detected.
- On the basis of obtained data, the contribution of H-bond among studied HIV-1 PR conformations might be prioritized as Ile50(B) > Ile50(A) > Asp25(A) > Asp25(B). It is also notable that none of the ligand binding conformations showed all of the four key H-bonds with HIV-1 PR while sixteen binding poses (1T7J, 1MUI, 1BV9, 1AJX, 1A9M, 1HPV, 2PQZ, 3O9H, 3SAC, 3SA9, 3MXE, 3SAB, 3MXD, 4DJR and 4DJO) interacted with three out of four residues simultaneously (Table 3).
- Analysis of binding maps showed that hydroxyl group of Amprenavir contributed to the H-bond(s) with Asp25(A) and Asp25(B) while sulfonamide oxygen atoms may be involved in H-bond interactions with Ile50(A) and Ile50(B) residues of HIV-1 PR. 2D schematic representation of binding interactions between Amprenavir and HIV-1 PR structure (PDB ID: 4DJO) is depicted in Figure 11.

*Regression analysis of docking results versus biological data*

Our regression analysis showed that docking outputs could be used for the elucidation of HIV-1 PR inhibitory activities (PDB bind database) with a relatively good predictability level (R^2^=0.703; Figure 5). Our results exhibited a lower regression coefficient for Binding MOAD activities (R^2^=0.443; Figure 6).

*Analysis of binding determinants*

**Figure 12:**
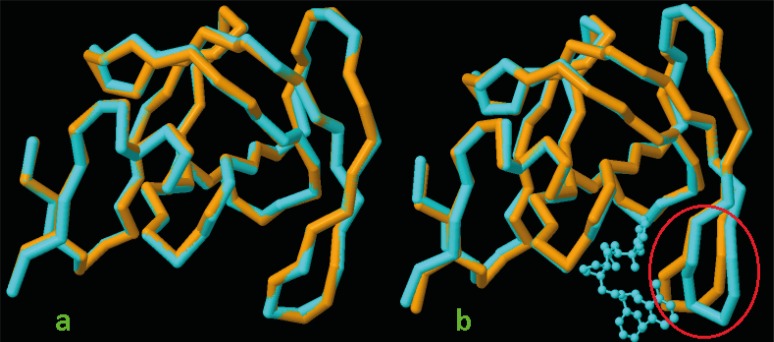
3D schematic representation of pair wise structure alignment between apo HIV-1 PR conformation (3IXO: orange stick) and a) 1HPV (RMSD=0.38 Ǻ) designated by blue stick, b) 1XL2 (RMSD=0.87 Ǻ) designated by blue stick containing a cognate ligand *i.e*., *N*-benzyl-2-(2,6-dimethylphenoxy)-*N*-[((3R,4S)-4 {[isobutyl(phenylsulfonyl) amino] methyl} pyrrolidin-3-yl)methyl] acetamide, the most distorted residues are highlighted by red circle in 1XL2

We decided to rank the probable determinant factors of the ligand induced enzyme conformation. In our opinion, the results of such study might assist in re-scoring the docking results within a screened dataset. Results of pair wise alignment study with apo conformation of the enzyme (3IXO) showed that co-crystallographic HIV-1 PR/Amprenavir complex (PDB ID: 1HPV) was associated with minimum geometrical deviation of enzyme from its apo structure (RMSD=0.38 Ǻ, Table 4). This observation confirmed the literature evidence that in binding to the inhibitors, a majority of enzymes might be necessarily redecorated via an optimum geometrical path (36). However, the most geometric deviation of the enzyme could be observed for the HIV-1 PR conformation designated by PDB code 2XL2 (RMSD=0.87 Ǻ, Table 4). For further consideration, 2D schematic representation of pair wise structural alignments between chains A of apo HIV-1 PR (3IXO) and holo HIV-1 PRs (IHPV and 1XL2) were depicted in Figure 12. Analysis of residues showed that maximum distortion of HIV-1 PR conformation in 1XL2 structure occurred within a loop containing Gly48, Gly49, Ile50, Gly51, Gly52 and Phe53 residues (red highlighted in Figure 12b). 

Estimated descriptors for various Amprenavir/HIV-1 PR systems (Table 4) were normalized (0-100%) to elucidate their probable significance in achieving the optimum target conformation upon binding to Amprenavir (PDB ID: 1HPV). For this purpose, each descriptor was designated by two numerical values indicating the optimum and non-optimum levels. In the case of ∆E_instability _of docked ligand conformation and RMSD of enzyme from reference structure, generally accepted optimum values were 0 kcal/mol and 0 Ǻ, respectively, hence these values were taken as optimum levels. It should be noted that no commonly accepted thresholds for optimum scores of AutoDock binding affinity, number of lipophilic interacted residues and number of H-bond interactions in a typical enzyme/inhibitor system could be rationalized. Due to this restriction, optimum levels of these factors were considered as the best achieved scores within the docked Amprenavir/HIV-1 PR systems. Similarly, the lowest numerical levels of all descriptors were taken as the worst achieved scores within the docked Amprenavir/HIV-1 PR systems (Table 5). The probable significances of five descriptors in the achieved induced fit of Amprenavir/HIV-1 PR complex (1HPV) were reported as significance percentages. Significance values of the descriptors were all estimated within the optimum and non-optimum levels (Table 5). 

**Table 5:** Levels of estimated factors for induced conformations of HIV-1 PR

**Descriptor**	**Non-optimum ** **level ** a	**Optimum level ** b	**Estimated for IHPV system**	**Significance% of the estimated descriptor for IHPV system**
AutoDock binding affinity (kcal/mol)	-6.64	-9.25	-7.83	45.6
Number of lipophilic interacted residues	11	23	19	66.7
Number of H-bond interactions	1	6	4	60
RMSD of enzyme from reference structure c (kcal/mol)	0.87 e	0	0.38	56.3
∆E_instability _d of docked ligand conformation (kcal/mol)	109.50 f	0	88.07	21.4

a *The worst achieved scores within the docked Amprenavir/HIV-1 PR systems (Table 4)*

b *The best achieved scores within the docked Amprenavir/HIV-1 PR systems (Table 4) except for last two records that are the generally accepted best scores.*

c *Reference structure is the apo form of HIV-1 PR (PDB code: 3IXO). *

d *∆E *_*instability *_*= E*_*top-ranked docked *_*– E*_*optimized conformation*_

e, f *Minimum level of these two descriptors is not translated into the lowest level, but it means the most inappropriate condition.*

Data mining showed that induced fit of Amprenavir/HIV-1 PR complex might be significantly determined by lipophilic contacts followed by deviation of enzyme from its native conformation, H-bond patterns, estimated free binding energy and deviation of ligand from its optimum conformation (designated by *∆E *_instability_), respectively. Of course we believe that such priority order have been achieved within the selected dataset in this study and more extended explorations through larger enzyme/inhibitor datasets would be less biased to the size of dataset.

*FP and FN*

Analysis of binding factors exhibited that none of the Amprenavir conformations could be recognized as FP points on the basis of factor d (conformational variation of enzyme from apo structure). This observation is very important and emphasizes on the determinant role of enzyme conformational variation in prediction of ligand induced binding poses. Further investigations via chemically diverse inhibitors may be possibly the subject of future investigations in this field. 

There was an opposite case for factor e (conformational variation of ligand from optimum structure); forty-two FP points could be predicted. Such a result may be translated into the uncertainty of factor e in prediction of HIV-1 PR targeted docking results and confirmed our previous results that inhibitors might not necessarily interact with the enzyme active site via their minimum energy conformation (18, 19). This was also in agreement with our above analysis on binding factors *i.e.,* significance percentage of 21.4% was estimated for factor e (Table 5). 

AutoDock binding affinities (factor a) and number of H-bond interactions (factor c) produced relatively balanced results (Figure 8). However analysis of docking results on the basis of hydrogen binding exhibited twenty-two non-FP/FN points. It should be noted that most of the H-bond patterns showed reasonable agreement with the binding pattern of Amprenavir in its crystallographic file (IHPV).

Most of the Amprenavir conformations were predicted as FNs on the basis of lipophilic interactions (seven FPs and thirty-seven FNs) but less non-FP/FNs were resulted (5 points). 

The outcomes of this study revealed that a major problem in docking based virtual screening is the proper selection of an enzyme conformation. Following this rationale and on the basis of results taken form ensemble docking approach, different scenarios may be considered: 

1) Docking validation (self-docking) protocols may be performed with less trouble due to the presence of induced target structure. 

2) Our ensemble docking approach on HIV-1 PR system demonstrated that varied binding results might be expected upon docking of a specific inhibitor (Amprenavir) into the multiple conformations of the enzyme. To alleviate the problem, a simple docking approach within an enzyme including a similar cognate (co-crystallographic) ligand (similar holo structure) followed by an efficient scoring function is proposed. 

3) In the case of holo enzyme structures bearing non-similar cognate ligands, an ensemble docking approach may be run by the cross-docking of a co-crystallographic enzyme inhibitor into the multiple enzyme structures (holo dataset). Subsequent analysis of probable induced fit determinants (section 3.1) may be done within the results of ensemble docking approach. Ranked induced fit determinants could be used in post-scoring of the ensemble docking results. 

4) When no holo structure is available, an ensemble docking approach may be run through apo structures of the enzyme. 

## Conclusion

Computer aided molecular design (CAMD) has spurred a renewed interest to deal with the growing body of information from genomic and proteomic efforts. In this regard, molecular docking is an attractive branch of CAMD that allows drug designers to simulate binding mode and predict binding affinity of different ligand-receptor complexes. In the present study, ensemble docking approach was successfully applied for modeling of anti-AIDS agent Amprenavir in the active site of HIV-1 PR. The outcomes of this study showed that success of a typical HIV-1 PR targeted docking strategy in rational drug design might be strictly depended on a selection of docked enzyme conformation. Further results showed that in selection of a desirable HIV-1 PR target for docking of amprenavir like ligands, lipophilic contacts are very important while the effect of ligand departure from its optimum conformation is less important. Pertaining to this, the multiple-receptors docking approach might be a suitable strategy to find a relatively optimum conformation of the enzyme to run the docking simulation of a query class of inhibitors. It is apparently known that our analysis method might be biased due to the restricted dataset of crystallographic files, but retrieved protein conformations (PDB database) can be regarded as valuable sources of such studies since they represent real induced enzyme conformations upon binding to the assayed inhibitors. Moreover; the results of ensemble docking approach may be complementary to molecular dynamics simulations and hence assist in finding optimum dynamic paths.
